# Being an older hospitalized patient during the COVID-19 pandemic - A qualitative interview study

**DOI:** 10.1186/s12877-023-04562-1

**Published:** 2023-12-05

**Authors:** Ann-Sofi Östlund, Julia Högnelid, Annakarin Olsson

**Affiliations:** Faculty of Health and Occupational Studies, Department of Caring Sciences, University of Gävle, Gävle, S-801 76 Sweden

**Keywords:** COVID-19, Experiences, Hospitalized, Older patient, Personal protective equipment (PPE), Qualitative

## Abstract

**Background:**

Older people hospitalized for COVID-19 are a vulnerable group due to the disease itself, aging and often loss of hearing and vision. Person-centered care, where patients have opportunities to communicate and participate in their own care, is important. However, because healthcare staff needed to wear personal protective equipment during the pandemic, to protect the patients and themselves, providing person-centered care was often difficult. This study aims to describe older hospitalized patients’ experiences both of being cared for, while having COVID-19, and of the care provided by healthcare staff wearing PPE.

**Methods:**

Fourteen older patients, over 65 years of age, were interviewed, and the data were analyzed using qualitative content analysis. The study adhered to Consolidated criteria for reporting qualitative research guidelines.

**Results:**

Three subthemes and one overall theme, *“The desire for survival overshadows difficulties”*, emerged in the analysis. The main findings revealed that the older hospitalized patients experienced the care they received from the healthcare staff as satisfactory. The older patients reported understanding and accepting that the pandemic situation meant that their ability to participate in their own care and communicate with healthcare staff were given lower priority.

**Conclusions:**

Older hospitalized patients need to be provided person-centered care, and situations such as a pandemic are no exception. Care tasks that are not acute in nature, e.g., planning for patients’ return home, could be conducted by healthcare staff not required to wear PPE.

## Background

Older people infected with SARS-coronavirus-2 (COVID-19) are at higher risk of becoming seriously ill and are often in need of extensive care. During the pandemic, healthcare staff had to quickly adapt to meet the need for care, while being forced to protect themselves and patients against the virus by wearing different kinds of personal protective equipment (PPE). The use of PPE has an impact on the communication and contact with older hospitalized people.

Nearly seven million people have died worldwide, due to or while infected with COVID-19 [1]. In Sweden the corresponding figure is around 17,000, and most of these people were between 60 and 90 + years of age [2, 3]. Older people were more likely to become seriously ill and to die from COVID-19 [4]. For most people with COVID-19, self-care at home is sufficient [5], but in some cases hospital care is required. If hospital care is needed, the patient must be isolated so as not to transmit the infection to other patients and/or healthcare staff and the healthcare staff needed to use PPE [6, 7], such as surgical masks, gloves, goggles, glasses, face shields, gowns, and aprons in the care of the patients. Patients infected with COVID-19 who have been cared for by healthcare staff wearing PPE have described both positive and negative effects [8] on the contact and communication between themselves and healthcare staff.

Isolation of hospitalized patients with COVID-19 has been used globally to decrease the spread of the corona virus. In previous studies, being cared for in hospital and being isolated has been shown to be a negative experience for patients, causing emotions such as fear, panic, helplessness, death anxiety and loneliness [9–12]. Furthermore, having other patients in the same room [11] as well as beeping monitors was also experienced as a distraction while the patient was in isolation [11]. It has also been shown that patients feared infecting others, which led to feelings of guilt [11, 13]. Such experiences may create difficulties in communicating with healthcare staff in a satisfactory manner [14]. The communication difficulties described were also due to patients’ perception that the environment was stressful for the staff [11, 14], language barriers and a feeling that staff were afraid of becoming infected if they spent too much time with patients [14]. Difficulties communicating with healthcare staff may also exist because the disease causes patients to lose control of their own body and begin to doubt their own judgment as well as the body’s signals [13].

Older people should be provided with person-centered care (PCC) [15, 16], and patients’ needs, and wishes should be considered. This means that, as far as possible, patients should be given opportunities to be involved in the decision-making, priorities, care, and treatments that are carried out to promote their health [17–19]. One prerequisite for PCC is thus good communication between healthcare staff, patients, and relatives [20, 21]. Older fragile people receiving hospital care want good communication with healthcare staff; this entails staff giving them information about their condition and listening to them [22]. According to older people, obstacles to good communication include e.g., hearing loss or other health problems [23, 24]. Such problems may mean they cannot participate by being part of the decision-making process to the extent they wished, particularly when too many people are involved in their care, healthcare staff are stressed, care sessions are short and there are language barriers [24, 25].

Very little is known about the consequences of being an older hospitalized person and being cared for by healthcare staff wearing PPE during the COVID-19 pandemic [8]. Gaining insight into older patients’ experiences of challenges associated with participating in their own care and communication, in accordance with PCC, with healthcare staff will give a broader picture of what factors may contributed to good PCC. Such knowledge is also important in enabling healthcare professionals to improve and individualize their support to older patients in the event of a new pandemic or other care that requires staff to wear PPE.

## Methods

### Aim

The aim of the present study is to describe older hospitalized patients’ experiences both of being cared for while having COVID-19 and of care provided by healthcare staff wearing PPE.

### Study design, participants, and setting

In the present descriptive qualitative study, semi-structured interviews were conducted between April and August 2021 in Sweden with fourteen participants, selected using convenience sampling. Due to the COVID-19 restrictions in effect at the time, interviews were held over the telephone. The target study population consisted of older people, 65 years of age or older, who had been hospitalized for COVID-19, cared for by healthcare staff wearing PPE and been discharged from hospital to their home for a maximum of three months. Exclusion criteria were experiencing cognitive decline, inability to communicate using spoken language or receiving palliative care. The participants were recruited from one regional hospital in central Sweden, on a ward where older people with COVID-19 were cared for. The hospital, one of six in the region, has several medical specialties (i.e., medicine and surgery departments for both adults and children, psychiatry, orthopedics, emergency and intensive care, childbirth, etc.) The ward where the participants in the present study were recruited has 12 rooms and 16 beds. During the day shift, a doctor with geriatric specialist skills, two junior doctors, three nurses, four assistant nurses, a physiotherapist and an occupational therapist worked on the ward. Two nurses and two assistant nurses worked the night shift.

The study involved six women and eight men (age range 65–83 years, median age 73.5 years). The participants were cared for at the hospital for a period of between two and 21 days, with a median length of stay of nine days. The PPE used by the healthcare staff were surgical masks, gloves, goggles, glasses, face shields, gowns, and aprons. The setting is a geriatric ward, an inpatient ward with isolation, and a gateway to each room. The patients were not allowed to leave the room, where oxygen support/treatment was available. No patients were intubated on the ward. If intubation was required, they were moved to a higher level of care (intensive care unit; ICU). In some cases, patients had previously been treated at an ICU, but when ICU care was no longer necessary, they were moved to the ward under study. In some cases, they were only treated on the present ward, but if their condition deteriorated and they needed intensive care, they could be transferred to a higher level of care (ICU). At the hospital, the patients were isolated so as not to spread the infection. Visitors were typically prohibited, but some exceptions could be made.

### Data collection procedure

The care planners (two registered nurses) on the ward were asked to assist in identifying suitable participants who met the inclusion criteria; after doing so, the care planners passed the information on to the author responsible for data collection (blinded for review). The author visited the ward and gave the potential participants verbal and written study-specific information and a written consent form, as well as a return envelope. Potential participants who agreed to be contacted by telephone for enquiry were informed that the author would call them approximately two weeks after their hospital discharge to ask whether they wished to participate in the study. For those who agreed to participate, a time for the interview was determined by the participant and scheduled, preferably within two weeks after the telephone call.

Starting from the study aim, an interview guide was created to maintain consistency in the format of the interviews. This interview guide consisted of questions/statements such as: *Can you tell us about your period of care at the hospital? Can you tell us about your experiences of being cared for by healthcare professionals who wore personal protective equipment? Feel free to describe a specific event. Tell us about your experiences of talking/communicating with healthcare professionals who wore personal protective equipment. Do you feel you were able to participate in making decisions about your care?* Responses were further explored using additional questions and probes. One pilot interview was performed to evaluate the interview guide for completeness, and if necessary, adjustments were made. After the pilot interview, questions regarding communication and participation were clarified. All subsequent interviews were performed by one author (blinded for review). The interviews lasted 35 min on average. Fieldnotes, notable in a telephone interview, were made to document, e.g., the person crying, laughing, or pausing.

### Data analysis

All telephone interviews were recorded on an Mp3 and transcribed verbatim. The text was coded, and confidentiality was ensured by using fictitious names for people and places in the transcriptions. Transcripts were analyzed using qualitative content analysis inspired by Graneheim and Lundman [26]. First, meaning units, words and sentences were identified in the interview that were relevant to the study aim. The meaning units were then condensed, hence reducing the amount of text. The condensed meaning units were then labeled with a code that described the text in a few words. This concluded the manifest part of the analysis [26, 27]. The codes were interpreted and compared for differences and similarities, and tentative subthemes were abstracted. The subthemes were then summarized in one overall thematization, based on the underlying meaning [26]. Reflections and discussions were conducted in the research group to reach agreement on the subthemes and main theme [28]. The analysis, thus described as a linear process, was performed as a back-and-forth movement between the whole and the parts [26]. The interview fieldnotes were used in the analysis to deepen our understanding of the data.

### Ethical considerations

The study was approved by the Swedish Ethical Review Authority (2021 − 01015) and implemented in accordance with the Helsinki Declaration. At the time of the interview, verbal consent was obtained, and the written consent was sent back to the author in the enclosed return envelope. There was no treatment relationship between the researchers and the participants prior to or after the study. All audio recordings, fieldnotes and coded data were saved on a password-protected server during the study project and only the researchers had access to the server.

## Findings

The analysis revealed three subthemes and one overall theme: *“The desire for survival overshadows difficulties”*. The theme and subthemes are presented in Table 1 and in the running text with additional quotes (informant x).

**Table 1:** Subthemes and theme

Subthemes	Theme
Perceptions of successful communication	*“The desire for survival overshadows difficulties"*
Surrendering to and trusting those who know	
Gratitude for the care provided	

The experiences expressed throughout the narratives – the older hospitalized patients’ disease state and their need for care – overshadowed other possible needs. Thoughts about how participation in care and communication worked when the staff were wearing PPE were of less importance: *“The people [healthcare staff] who are best at this and know how to act and behave in this situation, so I think it’s pretty easy to accept it, I think”* (informant 12). The participants had enough trust in the staff to surrender themselves to them, because they needed care to survive. As one participant said: *“They [healthcare staff] know what I need and I trust them 110%… I do… so I don’t know if I could question how they care for me… no, because I don’t know how I would care for myself”* (informant 8). Another participant said *“I listen to them completely… I’ve gone to the hospital to see people who can care for me… I trust in what they say”* (informant 10).

### Perceptions of successful communication

The participants reported that establishing contact for communication and identifying the staff wearing face masks was mostly accomplished by the staff introducing themselves by name and title when they entered the room or changed shifts or by looking at the name tags on the staff’s clothes. One participant said: *”the nurse came in and told me when they changed shifts so I would know who was who [among the healthcare staff]”* (informant 8). It was also possible to identify the staff by their work tasks or through the window when they were not wearing PPE. The participants did not experience identification of staff as a problem. When the name tag was missing or covered, they just asked who the person was. Even if a staff member had introduced him-/herself previously, the participants sometimes had difficultly remembering the name owing to their own poor memory.

Most of the participants did not experience any obstacles to communication when the staff wore face makes, but felt it was the same as without PPE. Although PPE affected communication in some respects, making lip reading impossible and speech less clear, especially for the hearing impaired, the participants still felt that communication was good. They were able to hear variations in mood, such as joy and laughter. The participants also experienced a good connection with the staff; they understood what the staff said, despite the PPE, but their disease affected them: *”I understood what they [healthcare staff] said … then how sick I was, that’s another thing”* (informant 2). As far as the participants knew, they also mostly understood all the information given, especially when staff stayed in the room, took their time and talked for a while. The participants also experienced that the staff understood most of what they said, but sometimes they were not sure.

The participants reported not understanding everything the staff said when the staff used medical terms or when a staff member was not a native speaker of Swedish and was wearing a face mask. Communication difficulties were simply resolved by repeating the question or writing it down om paper: *”Sometimes I didn’t hear what they said, but then I just asked and they told me again”* (informant 7). Furthermore, the participants reported accepting possible communication difficulties given the circumstances, meaning the staff needed to use PPE: *“Sure it was a bit difficult to hear, but it was necessary [for staff to wear PPE]”* (informant 10). The participants felt the staff did their best to facilitate and/or adjust the communication. They also reported that the staff seemed to have experience communicating with older patients and adjusting communication to the “*right level”* (informant 3). The staff were perceived to be *“thinking about me”* (informant 5), which was considered the most important.

### Surrendering to and trusting those who know

The participants reported having been given opportunities to participate in the decision-making and planning of their care, and they did not experience any obstacles to doing so. They felt that the staff involved them by telling them what they were doing now and planning to do and what treatment they were planning to give. The staff asked the participants how they were feeling and the staff listened to them:” Yes, *they [healthcare staff] constantly explained what they were going to do and were doing, it was fine, everything, nothing out of line”* (informant 7). The participants also felt they had been given opportunities to participate in the decision-making regarding their care when staff encouraged them to ask questions and in other ways express their wishes. They also felt involved when they could decide what to do, for example, whether they wished to eat, rest, change rooms or positions/bed settings as well as when they were not forced to do anything. Although some participants did not always experience being involved in the decision-making or being able to make choices about the care or treatment presented to them, they nevertheless described trusting in the staff’s knowledge and willingness to help. For these reasons, the participants were willing do what they were told and did not question staff decisions. One participant said that the caring staff: “*knew what was best and knew the most, better than I do and I went to the hospital to get help and was grateful for any care I got”* (informant 13).

Some participants mentioned that they, on some occasions, would have liked the staff to provide more information. They would have appreciated knowing how they could increase their participation in the decision-making, change rooms or wards, when and if sharing a room was likely and if, when and how discharge was planned. They did not like being able to decide at what time they, for example, would take a shower. On the other hand, some participants reported not wanting to know all the options or be more involved or not knowing whether they were capable of being more involved; they were satisfied with the care provided. Others reported not always knowing whether their own wishes and decisions were considered, but also had not given it much thought.

The participants were cared for in either single or multibed rooms. Because the patients were in isolation, the staff could not always be present in the room. Regardless of whether the staff were present in the room, the participants experienced them as being available. The participants reported that the staff made time for them while in the room, often staying and talking for a while, which made the participants feel listened to. If they needed help or had any questions, they used the alarm button at their bedside and, in their experience, the staff were quickly in place in response to their call:*”If you had questions you could press the alarm button”* (informant 5). Some participants reported not always having opportunities to ask their questions, as the staff only stayed in their rooms for a short time. Sometimes the participants also experienced the staff just walking by when they were trying to get their attention by waving through the windows to the hallways. The participants also reported not wanting to bother the staff and waiting to ask or doing the task themselves. Situations when the participants had opportunities to ask spontaneous questions were during the physicians’ rounds and when medications were distributed by the staff.

### Gratitude for the care provided

The participants reported that the COVID-19 infection affected their state of health to such a degree that they sometimes not remembered details from their hospitalization. Several of the participants had earlier experiences of being hospitalized, but had never experienced anything like this disease: *”I have never in my life been as sick as this… and I’ve had a heart attack and things, but this was worse”* (informant 2). The participants said they accepted all the help they were offered, given how ill they were. They also said they accepted being at the hospital, because they would not have survived without hospital care.

The participants felt the care they received was critical to their survival, and they were very grateful to the healthcare staff. They also said the staff were “*amazing, polite and helpful angles*” (informant 8) as well as highly committed, accommodating, and conscientious in their work. The participants were very satisfied with the care they received and felt the caring staff had their “*best interests*” in mind (informant 14). They felt safe and believed the staff had the knowledge and competence required for their care. Furthermore, the participants expressed admiration for the staff for what they must put themselves through and for their ceaseless effort to encourage them when they did not always have the strength. They also reported that the staff made their period of hospitalization a positive experience, despite the difficulties associated with the disease. One participant said: *”The staff are so good, unbelievably good and they must be exhausted but still so happy and considerate of their patients, because I’m sure many patients are difficult”* (informant 10). Some participants mentioned that all staff had “*a smile on their faces”* (informant 14), even though they must have been exhausted due to their heavy workload.

According to the participants, it was the care they received that allowed them to survive the disease: *“What about the care was I specifically pleased with… everything… they saved my life”* (informant 3), and they fully understood why the staff needed to wear PPE while caring for them. Most participants knew about PPE before hospitalization, through the media and from relatives working in healthcare: *”Well, I didn’t think so much about it [PPE] more than what I’d seen on TV all the time”* (informant 2). Some had been informed about it directly upon arriving at the emergency ward, and others reported not having reflected on it. The participants said they realized the staff were wearing the equipment to protect themselves as well as the patients. They appreciated knowing that the virus was not being spread. The participants reported not experiencing any differences in the care provided compared to previous hospitalizations, when PPE was not necessary. One participant said: *”I didn’t have any problem with it [the care] at all, it worked like normal but it was just that they wore face masks…. And a few had these rubber masks over their mouth, but no… I didn’t find it odd at all”* (informant 5). All participants understood that the staff had to wear PPE and accepted it, stating that it could not have been any other way: *”There was nothing strange about it [the PP]… of course I understand that, if I’m lying there getting wonderful service, then they want to protect themselves, otherwise we’d have to share a bed… all lying there together ”* (informant 14). Some participants, however, found the PPE a bit strange and frightening, when only eyes were visible, but they got used to it. One participant said that it *“made associations to war”* (informant 4) due to the breathing gear. Some also reported feeling more exposed to the virus when the staff did not wear face masks, such as when the participants were being transported to other parts of the hospital for different examinations (e.g., X-ray). Furthermore, the participants expressed empathy for the staff who had to wear the PPE, because they saw them sweating, having trouble breathing, and trouble seeing due to condensation on the glasses attached to the mask or on a visor.

Most experiences of care provided were described by the participants as positive. However, one worry while at the hospital was whether they would need to share a room with other patients. As one participants said: *”There were two of us lying there, if I passed the screen I was inside the other person’s private sphere”* (informant 4). They did not want to disturb the other patient by, for example, passing the screen between them. Moreover, the screen was blocking the view from the window. The other patient could also exhibit disturbing behavior, such as speaking loudly and often on the telephone. Furthermore, the other patient’s condition could be worrying, if the participants noticed that he/she was very sick or that his/her survival was not certain. Other participants said they understood the situation (the need to share a room) and did not experience it as a problem. Some even reported appreciating the social aspect of sharing a room, provided they got along well with the other patient. Some participants felt that the isolation necessary to avoid spreading the disease was unpleasant, boring, and made them feel trapped because the room was small. Overall, being isolated was experienced as acceptable, but above all as necessary and understandable, natural, and not a problem. Some even appreciated the peace and quiet and the opportunity to decide on their own, for instance, what TV channel to watch.

Some participants reported that changing rooms and wards several times during hospitalization was disturbing, and they considered many of the changes unnecessary. Some also described feeling worried when they felt the staff were lacking in knowledge, for instance if a staff member seemed insecure when handling the oxygen equipment. Another disturbance mentioned was that treatment of other ongoing health problems was delayed owing to the limited space in the room. Another worry concerned when they were supposed to be discharged from hospital, was how things would work when they came home and whether enough preparation time would be given at the hospital to adjust to the situation.

## Discussion

The present study showed that the older hospitalized patients were mostly satisfied with the care provided by the healthcare staff. Their experiences of being involved in decisions regarding their own care and communication were of lower priority, given the help and support they received from the healthcare staff, despite them wearing PPE. Communication was sometimes described as a problem when staff wore PPE, however, this too was over-shadowed by the pandemic situation.

Older people may suffer from various degrees of cognitive decline, due to sudden and/or temporary illness [4], thus such decline was an exclusion criterion in the present study. Previous research has shown that older people’s participation in decisions regarding their own care is very important to strengthening and supporting, e.g., individual autonomy [17, 29]. However, in the present study, the older patients’ ability to communicate and be involved in making decisions about their own care was thought to be of less importance compared to the help and support they received from the healthcare staff. Wu et al´s., [30] study, involving hospitalized older persons, showed that they were generally satisfied with the care provided, something also found in the present study. Studies have shown that patients wished healthcare staff would have provided them with more information about their condition [11, 31] and that patient sometimes had doubts as to whether the treatment they received was tailored to them [11]. The nurses caring for the patients also experienced that, when patients received information about their condition, they were more likely to follow the nurses’ instructions [31]. Data for the present study were collected during the second wave of the pandemic, which may have increased the participants’ knowledge, awareness and understanding of the severity of COVID-19, thus affecting their experiences. In contrast, one study [32] showed that older people felt they had lost their dignity, autonomy and “sense of self” during hospitalization for COVID-19. Healthcare staff therefore need to pay attention to older patients’ ways of expressing such feelings.

The significance of patient participation in nursing care has been described in several previous studies [33–35], and nurses also need to ensure older patients’ participation, in accordance with PCC [16]. However, in the present study, the older hospitalized persons felt that being able to participate was less important than surviving the COVID-19 disease. It is more difficult for patients to engage with staff who are wearing PPE, which acts as an additional obstacle to communication [36]. Physical barriers such as face masks/visors may make it difficult for patients to communicate with healthcare staff [37], and even more difficult for patients with hearing and/or vision impairment [38]. According to older people, participation meant being a co-creator of their own care; it was founded on being treated with sensitivity and support, being told what was going to happen, taking responsibility, asking questions and being able to influence care [33]; many patients also felt that decisions were made for them prior to or during hospitalization. Working in PPE was also reported by healthcare staff to negatively affect communication between them and the patients [38, 40].

In the present study, being placed in a shared room with other patients was experienced both negatively and positively. Some experienced their private space as limited and were worried they might disturb the other patient by making noises (e.g., watching TV or talking on the phone). They also reported that “*the seriousness of the disease (COVID-19) was more obvious*” if the other patient was very ill. Others felt sharing a room gave a feeling of social togetherness. Previous research [41, 42] has shown that many patients prefer a single room, referring to the benefits of increased privacy, reduced noise (from both the other patient and staff caring for him/her), improved sleep quality, preserving patients’ privacy and autonomy to achieve greater control over their environment, and better communication with staff and healthcare workers. In contrast, a newly published systematic review by Bertuzzi et al. [43] showed that older adults preferred a shared room to avoid feelings of loneliness. The amount of time since the outbreak of the pandemic may have led to this result, thus making a shared room an opportunity for social interaction with others.

Nursing staff have stated that they lacked the training needed to care for patients with COVID-19 [44, 45], had an excessively high workload and that they needed to manage a situation they had never been in before. They often had to work with a shortage of staff and insufficient PPE [46]. Healthcare professionals have also stated that they were afraid both of being infected by the virus themselves and of infecting their relatives [31, 45, 47]. Studies, including healthcare staff wearing PPE (or performing various kinds of barrier care) while caring for older patients suffering from, e.g., Methicillin-resistant Staphylococcus aureus (MRSA), has shown that isolation may result in negative psychological effects including anxiety, stress, and depression, but may also result in patients receiving less or substandard care [48]. Personal protective equipment may also constitute physical barriers to effective communication with patients in isolation [49]. Even if isolation in many situations may be experienced as negative and intrusive, Shaban et al. [14] found, as in the present study, that isolation was appreciated because it gave greater trust in healthcare staff and their method of providing care.

### Study limitations

One strength of the present study is the uniqueness of describing older hospitalized patients’ experiences both of being cared for, while having COVID-19, and of the care provided by healthcare staff wearing PPE. A heterogeneous group of participants was included in the study owing to the use of convenience sampling. Thus, the study findings need to be interpreted in relation to some methodological considerations. First, because the results concern a small number of older people from one hospital in Sweden, they may not be transferable to a global population. However, the number of participants was adequate for the purpose of the present qualitative study. Moreover, the insights gained, and experiences reported are likely to be similar to those of other older hospitalized people cared for in other settings by healthcare staff wearing PPE. Another limitation that must be considered is the fact that additional attributes, that is e.g., medical information about the participants, may have increased the understanding of the results. The interviews were planned as soon as possible after the participants had been discharged from hospital, taking into account that they must be given sufficient time to recover. A weakness of the study may be that, of the 24 older patients invited to participate, only 14 agreed to do so. We interviewed the participants some weeks after discharge. This meant participants had to recall their experiences, which might have been difficult after having had COVID-19. Rather than this indicating a low rate of willingness to participate, it may reflect the problems experienced by this group during the pandemic. There is also a possibility that older patients who had a strongly negative experience of their hospitalization are unintentionally excluded in the present study. A way to overcome this limitation might have been to do a questionnaire survey, including all of the inpatients.

One researcher performed all interviews. To establish credibility, all authors read all the interview transcripts. Thereafter, the first and second author discussed the steps in the analysis to the level of codes, and then from the level of subthemes to overall theme, a discussion was held among all authors. Consensus concerning interpretation of the participants’ experiences was achieved among all authors, assuring dependability. Data were collected by following an interview guide. To further strengthen credibility, quotes from the interviews were used in presenting the results. The Consolidated Criteria for Reporting Qualitative Studies (COREQ) checklist [50] was used as a guide to reporting the study.

## Conclusions

The COVID-19 pandemic is set to continue to impact the way healthcare is delivered for the foreseeable future. The study revealed the importance of understanding how older hospitalized patients’ experiences both of being cared for, while having COVID-19, and of the care provided by healthcare staff wearing PPE, hence the results play a significant role in improving care for older patients and provision of PCC. The fact that staff must always wear PPE in care encounters may entail that non-emergency care, for instance planning for patients’ return home, might be neglected or not provided. A care coordinator might be employed to work with less acute tasks (that do not require healthcare staff to wear PPE).

## Data Availability

To protect participants’ privacy and confidentiality, the data (transcripts) generated and analyzed for the present study are not available, but will be available from the corresponding author on reasonable request.

## Declarations

The study is performed in accordance with the Declaration of Helsinki. Written informed consent was obtained from the participants.

## Abbreviations

ICU: Intensive Care Unit
PCC: Person-centered Care
PPE: Personal Protective Equipment
COREQ: Consolidated criteria for reporting qualitative research
WHO: World Health Organization
